# From data to analysis: linking NWChem and Avogadro with the syntax and semantics of Chemical Markup Language

**DOI:** 10.1186/1758-2946-5-25

**Published:** 2013-05-24

**Authors:** Wibe A de Jong, Andrew M Walker, Marcus D Hanwell

**Affiliations:** 1 EMSL, Pacific Northwest National Laboratory, P.O. Box 999, Richland, WA 99352 USA; 2 School of Earth Sciences, University of Bristol, Wills Memorial Building, Queen’s Road, Bristol, BS8 1RJ UK; 3 Department of Scientific Computing, Kitware, Inc., 28 Corporate Drive, Clifton Park, NY 12065 USA

**Keywords:** Chemical Markup Language, FoX, NWChem, Avogadro, Computational chemistry

## Abstract

**Background:**

Multidisciplinary integrated research requires the ability to couple thediverse sets of data obtained from a range of complex experiments andcomputer simulations. Integrating data requires semantically richinformation. In this paper an end-to-end use of semantically rich data incomputational chemistry is demonstrated utilizing the Chemical MarkupLanguage (CML) framework. Semantically rich data is generated by the NWChemcomputational chemistry software with the FoX library and utilized by theAvogadro molecular editor for analysis and visualization.

**Results:**

The NWChem computational chemistry software has been modified and coupled tothe FoX library to write CML compliant XML data files. The FoX library wasexpanded to represent the lexical input files and molecular orbitals used bythe computational chemistry software. Draft dictionary entries and a formatfor molecular orbitals within CML CompChem were developed. The Avogadroapplication was extended to read in CML data, and display molecular geometryand electronic structure in the GUI allowing for an end-to-end solutionwhere Avogadro can create input structures, generate input files, NWChem canrun the calculation and Avogadro can then read in and analyse the CML outputproduced. The developments outlined in this paper will be made available infuture releases of NWChem, FoX, and Avogadro.

**Conclusions:**

The production of CML compliant XML files for computational chemistrysoftware such as NWChem can be accomplished relatively easily using the FoXlibrary. The CML data can be read in by a newly developed reader in Avogadroand analysed or visualized in various ways. A community-based effort isneeded to further develop the CML CompChem convention and dictionary. Thiswill enable the long-term goal of allowing a researcher to run simple“Google-style” searches of chemistry and physics and have theresults of computational calculations returned in a comprehensible formalongside articles from the published literature.

**Electronic supplementary material:**

The online version of this article (doi:10.1186/1758-2946-5-25) contains supplementary material, which is available to authorized users.

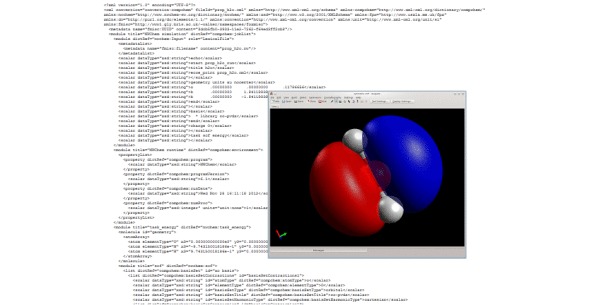

## Background

In chemistry, the key to successful multi-disciplinary integrated research is oftenthe ability to couple the diverse sets of data obtained from a range of complexexperiments and computer simulations to solve scientific problems that areintractable when only one technique is employed. In an ideal world any researcher inany discipline should be able to easily access, find and synthesise all thescientific data pertaining to the scientific question, molecular system or materialbeing studied [1]. With this data the researcher has a knowledge base that has thepotential to deliver new unexpected insights when all the information is broughttogether. Access to all scientific data relevant to the study at hand can also avoidrepetition of previous experiments or simulations, and can serve as a starting pointfor generating new ideas for the design of alternative new approaches ormolecular/materials systems, or can provide a framework for validation [2]. Increasing quantities of detailed data is gradually being made availableto scientific users in the scientific literature [3], and more widely in data repositories and other systems [4].

Raw data (for example the recorded NMR signal or calculated molecular orbitals) isonly a subset of all the scientific data generated from an experiment or computersimulation. This acquired data gets processed and analyzed with a barrage of toolsto extract the important observables, i.e. derived data, and help create thescientific interpretation. This derived data is most likely stored in theresearcher’s notebooks and sometimes forms part of scientific publications andpresentations, but it rarely gets used to annotate the raw data. Observables are thecommon language at which computational chemistry and experimental communitiesinteract, and these should be accessible as part of the scientific dialogue to makeavailable data useful to the scientific community at large.

A key challenge is the need to remove technical barriers to access scientific data,and common data formats play a key role in this respect. In the experimentalcommunity raw data is often stored in a standardized format once it has beenacquired (e.g. NeXus [5] or Scientific Data Exchange [6] using HDF5 [7]), lightly annotated with details of the experiment itself. While theexperimental community has been working to develop and deploy data standards, thisis less true in the computational chemistry community.

Historically, complete data sets from computer simulations, including input and allgenerated ASCII and binary output data files, have not been made widely available.These files are not generally stored in accessible data repositories or included assupplements with publications. Within computational chemistry and materials sciencethere are multiple efforts to make a limited set of calculated observables availableto the broader community. Examples include the Materials Project at MIT [8], CatApp at Stanford [9] the Computational Results Database at Washington State University [10] and the Computational Chemistry Comparison and Benchmark Database at theNational Institute of Standards and Technology (NIST) [11]. The latter also links the information to the available experimental dataavailable at NIST. Each of these efforts provides easy access to commonly usedobservables using diverse and non-standard data formats, but often with incompletescientific data sets or lacking the important meta-data.

The key to effective integration, mining, reuse, and visualization of diverse datasources is to work within standardized and semantically rich data formats. Themyriad of simulation data also stifles advances in the development of open-sourcedata mining/search tools and visualization tools (such as Avogadro [12]) due to the significant efforts to develop and maintain translationinfrastructures for all the data formats. A good example of standardization is theCrystallographic Information Framework, which includes the widely usedCrystallographic Information File (CIF) [13] maintained by the International Union of Crystallography (IUCr).Naturally, many of the more recent efforts to develop standardised data formats havemade use of the extensible markup language (XML) to define the format. The variousXML standards provide a widely implemented framework for defining the basic syntaxof data files, mechanisms for specifying the permitted structure and content of suchfiles and common approaches to reading, writing, manipulating, normalising andvalidating standardised documents. These standards and tools enormously simplify thetask of designing and deploying sharable data formats. Some relevant examplesinclude the work of Gygi and co-workers, who developed a rudimentary XML-basedstandard for the interchange of simulation data [14] that is used in their framework for the validation and verification ofelectronic structure codes, called ESTEST [15]. Researchers at NIST led an effort to develop an XML standard for theinterchange of materials information (MatML) [16].

Perhaps the most successful attempt to standardize the development of common languagefor chemistry is the Chemical Markup Language (CML), developed by Murray-Rust andRzepa since 1995 [17–21]. CML provides the vocabulary needed to express a very wide range ofchemical (and related physical) concepts in an XML document. This vocabulary isspecified in an XML Schema definition (XSD) document that is deliberatelypermissive: it must be as the use of valid CML documents is extremely varied [22, 23]. CML documents can act as interactive scholarly manuscripts [21], as supplementary data to support more traditional publication [3], as the primary data format for a range of experimental and computationalstudies [2, 24], or as a means of data exchange within automated workflows [25–28]. For some of these applications it can be useful if the documentstructure and content is further restricted with additional constraints beyond thoseenforced by requiring validity as defined by the CML XSD specification. Theseadditional constraints, termed CML Conventions, provide discipline-specific meaningto CML documents and reduce the development burden for document consumers andproducers [29]. CML dictionaries provide additional fine-grained semantics with specificconcepts identified by terms referenced from elements within CML documents [29]. Furthermore, these mechanisms impose additional good practice ondocument produces by requiring the inclusion of defined metadata to preserveprovenance, and by insisting that all numerical data carries a machine readablespecification of its units. It is the ability to impose strict syntax and definedsemantics, and to validate these using well-understood tools, that means XML ingeneral and CML in particular is still the tool of choice for the storage andexchange of scientific data. While we imagine that alternative data representations,such as the JavaScript Object Notation (JSON), may be used in this context forefficient data exchange we do not foresee the development tools for powerfulvalidation and semantic transformation that motivate the use of XML, as discussedbelow.

To enable different computational chemistry codes to interoperate it is key that allessential data is stored in a common format. Within the computational chemistrycommunity various simulation codes, for example, MOLPRO, VASP, and Quantum-Espresso [30–33], have been adapted to produce a code specific XML output. Other projectshave utilized components of CML as an enabling tool for data exchange, storage andprocessing. The Quixote project seeks to build a collection of tools to allow datafrom computational chemistry calculations to be stored, shared, organised andqueried [25]. Key to this effort is the creation of a CML document for eachcalculation. This is then ingested into, and processed by, an instance of theChempound database system. By contrast, the eMinerals [34] and Materials Grid [35] projects sought to develop automated scientific workflows forhigh-throughput computation in atomic-scale mineralogy and materials science. Theseworkflows combined distributed grid computing [26, 27] with tools to generate input files, extract and analyse output data, andcreate and store key items of metadata [28]. An important aspect of this work was the generation of a representationof the key data in a CML document. The approach taken to allow these documents to beeasily produced on the myriad of systems that formed the distributed computingenvironment utilised by these projects was to directly generate CML as thecomputational chemistry application was executed. As the majority of suchapplications are written predominantly in Fortran, and the installed software on thecomputational resources was so varied, this involved the creation of a pure FortranXML library called FoX [36, 37], described below and in more detail by Murray-Rust and co-workers inanother article in this special issue [38].

An alternative approach would be to utilize Python or a similar high level languageto wrap the Fortran code and use features of the high level language to generate theCML. However the integration of Python and Fortran codes requires quite intrusivechanges to the Fortran application including the development of interface routinesfor each piece of functionality in the code that needs to store output data in theCML document, a redesign of the existing build and test system, and potentially achange in the skills needed by members of the developer community. While we considerthat this may be a viable approach if integration into a high-level programmingenvironment was being undertaken for other reasons, we avoided this change merelyfor the purpose of generating an XML document. As new concepts, conventions, anddictionaries are defined within CML, new common interfaces can be developed in theFoX library and used by the computational chemistry codes. FoX and its interfaceswere used to allow solid-state simulation codes such as SIESTA [39] and GULP [40] to directly output CML without introducing additional dependencies intothe applications’ compilation process. Very recently, developers of theTURBOMOLE package reported the use of CML and the interfaces in the FoX library tostore a subset of their output data needed to develop a database for computationaldata [41].

In this paper the further development of a FoX based infrastructure to producesemantically rich CML documents with the NWChem computational chemistry software [42] will be described. NWChem is one of US Department of Energy’sopen-source computational chemistry software packages, developed at theEnvironmental Molecular Sciences Laboratory (EMSL) National User Facility located atPacific Northwest National Laboratory. In addition, some developments of the CMLlanguage for computational chemistry will be discussed in some detail. Furthermore,the reading, ingestion and visualization of the CML documents generated by NWChemwill be demonstrated in Avogadro, an open-source, cross-platform molecular editorand analysis tool written mainly in C++ [13].

## Methods

### Generating semantically rich data with NWChem

The NWChem software like many other computational chemistry software applicationsproduces various data files during a simulation. These data include a humanreadable descriptive output file and binary files potentially containing fromtens of megabytes to multiple gigabytes of additional data (such as molecularorbitals and molecular dynamics trajectories). One approach to the creation of aCML document to describe an NWChem calculation is to post-process the existingoutput data files and convert these into CML. This is the path taken by theQuixote project where Java Universal Molecular Browser for Objects (JUMBO)converters are used to transform the human readable output files to CMLdocuments that can be digested by tools such as Chempound [25]. The major disadvantage of the use of converters is that they need tobe continuously modified and maintained because the output files they read havea tendency to change, for example when new functionality is added to NWChem. TheNWChem developer modifying the codebase in this way has no way of knowing if thechange causes external tools to fail and, probably, has no reason to care. Inaddition, the content extracted from the simulation is limited to what iscontained in these text based output files. Additional information held withinassociated large binary files, for example molecular orbitals or time evolutiondata, is lost unless this data also gets post-processed. Both of these issuesare addressed by our approach as described below.

In addition to the text and binary files, NWChem has a built-in infrastructure toproduce an additional ASCII file that contains key-value pairs. This file servesas input for the EMSL developed Extensible Computational Chemistry Environment(ECCE) graphical user interface and visualizer [43]. Ironically, ECCE reads this file and translates it into anapplication specific XML format that is used internally. This means that boththe JUMBO converters and ECCE use multiple steps to obtain a similar final XMLproduct.

As part of the current work, a prototype CML writer capability in NWChem has beendeveloped that writes a CML file during the simulation run. To minimiseintrusive modifications to the code base, the implementation builds upon theinfrastructure in NWChem for writing key-value pairs into the ECCE ASCII file.This infrastructure inherently already opens a data file at start up, writesdata to the file, and closes the file when the simulation ends. Instead ofwriting a standard ASCII text file, NWChem’s ECCE infrastructure has beenmodified to make use the FoX library to create the CML document. This makes moredata available to the CML writing machinery than is written to the output filedesigned for human consumption. We have benchmarked the performance impact ofwriting CML files on a larger (6 minute runtime) version of the example caseused in this paper and find the run time to be increased by less than 0.5%(the impact falls below 0.1% for even larger calculations). The CML writingcapability is being integrated into the main NWChem development tree and willbecome a standard output format in a future release.

**Figure 1:**
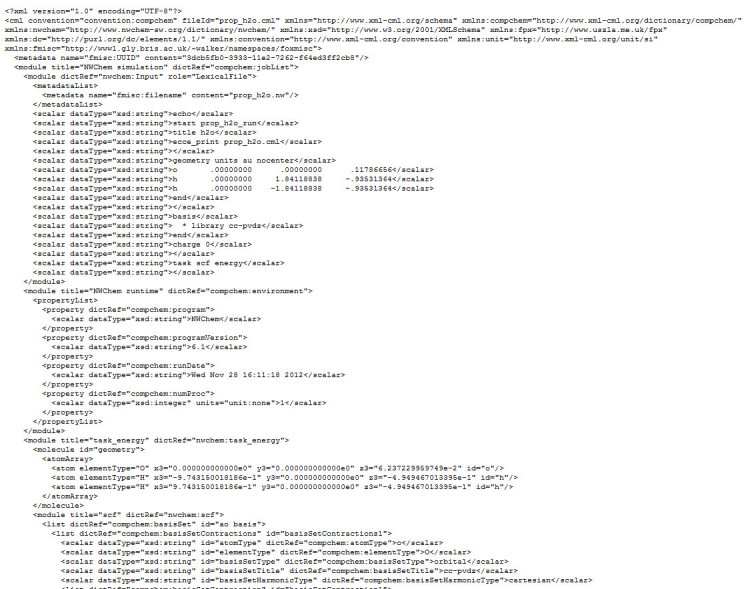
**First section of CML validated XML data, generated with NWChem usingthe FoX library.**

As discussed elsewhere in this issue [38], the FoX library permits the reading, writing and manipulation ofarbitrary XML documents in a Fortran-only environment. As expected, FoX will notpermit the creation of an XML document that is not well-formed. As well as thegeneral-purpose interfaces, FoX_wcml provides a specialised mechanism forgenerating CML output tailored to computational chemistry applications. Thisinterface, which was used for the majority of the CML generation from NWChem,does not attempt to allow the creation of any arbitrary CML document but insteadfocuses only on the needs of the majority of expected use cases in computationalchemistry and to a large extent imposes adherence to the relevant conventions.For example, users of FoX_wcml are not able to add atoms to a molecule withoutspecifying both position and element name, avoid declaring (and using) therelevant namespace for CML, or introduce numerical quantities without specifyingunits. Data passed into the FoX_wcml interface is accepted in a format expectedto be present inside of computational chemistry applications but some dataconversion is still sometimes needed. Figure 1 showsan example of a fragment of the CML document that can be generated from NWChemwith this implementation. In Additional file 1 weprovide a full example of a CML document produced by our current implementation.We note that this passes the tests implemented by the CML validation service [44].

A side effect of the constrained interface is that new ideas for therepresentation of output data cannot easily be generated with FoX_wcml. However,the generic FoX_wxml interface can be used in concert with FoX_wcml to produceadditional output in an already open CML document. We made use of this featureto expand the FoX_wcml interface to include an ability to write molecularorbitals to the CML document. Molecular orbitals are currently not well definedin the CML language. Appendix 1 outlines a draft CML computational chemistrydictionary for molecular orbitals, and shows a formatted example that can beused as a template to develop a molecular orbital convention in the future. Wealso enabled the ability to include a representation of the main NWChem ASCIIfile input parameters within the output document. Such ‘echoing’back of the exact code input in the output stream is very common incomputational chemistry and is essential to preserve data provenance (forexample, if the input files are inadvertently lost or modified). An example ofthe data inside a CML document produced by NWChem is provided inFigure 1 and a more formal definition of theproposed CML format echoing computational input is included in Appendix 2 (wherewe assume only ASCII data will be stored: arbitrary binary files with, forexample, wave functions are out of scope). This new functionality in the FoXlibrary is publically available in the development source control system andwill be included in the next formal release of the software.

### Visualisation and analysis of semantically rich NWChem data with Avogadro

In order to read in the CML produced by NWChem or any other computationalchemistry code, a new reader was developed for Avogadro in the OpenQube library.As discussed briefly in another publication in this journal issue [12], OpenQube was developed to address the requirements of normalizationof data produced by codes employing Gaussian type orbitals for calculations. Itwas already able to recognize the data available in output from several otherquantum codes, with the new computational chemistry CML reader being one of thefastest to develop using the compact PugiXML library as a lightweight XMLparsing engine. The example NWChem CML data in this paper was read into Avogadrousing the new reader, and from that point on behaved as other data files.

**Figure 2:**
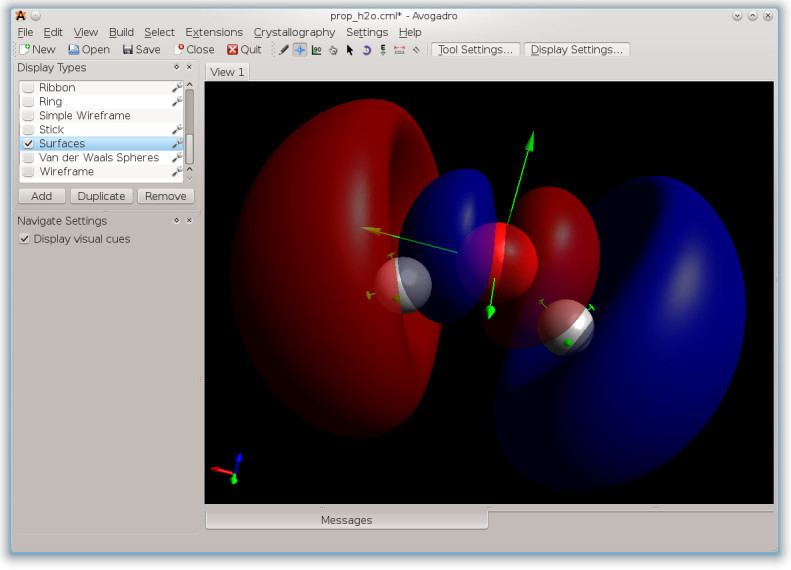
**Visualization of molecular orbital isosurface calculated by Avogadrousing the NWChem CML data, with NMR tensor projections andmagnitudes represented as arrows.**

An experimental branch is available for Avogadro that can read in importantelectronic structure information from NWChem, display it to the user and evenproduce further input for NWChem based on geometries from previous calculationresults. This allows Avogadro to leverage the semantic information stored in theCML document with minimal code changes. Figure 2shows an integrated visualization of the molecular orbital isosurface and thenuclear magnetic resonance (NMR) shielding tensors calculated by Avogadro usingthe data read in by this new reader, successfully demonstrating an end-to-endsemantically enriched workflow. The visualization of both properties in morecomplex molecules can provide new insights into molecular bonding.

The new reader was written using the PugiXML C++ XML parser library, and took theapproach of using an efficient Document Object Model (DOM) to represent the CMLdocument, and specific functions to read in and process XML nodes of interest.One of the major advantages of this approach over other readers was the minimalamount of parsing code as it was possible to rely upon the standard XML parsingroutines to find the nodes that contained data that needed to be read in. Morework will be required to generalize the approach taken as a single CML documentcan contain a rich computational experiment with multiple steps. Additionalinterfaces must be developed in Avogadro to go beyond a simple view of an outputfile containing one result to that of a rich document that can contain multiplesteps with links.

A major advantage of the CML reader is that of extensibility; where the CMLdocument can contain elements that this version of the reader does notnecessarily understand. Most other readers are somewhat fragile, and requireupdates when the computational code adds extra elements to its output. Thisreader is able to scan the CML document for the tags and attributes itunderstands, if more tags/attributes are added in the future the reader willstill be capable of understanding the document, with updates to the readeradding support for the new elements. This allows for the development of a muchmore robust approach to reading and transforming the data in the Avogadroapplication that was simply not possible in the other readers developed.

## Results and discussion

Our experience of the use of FoX for introducing CML output into NWChem hasreinforced our understanding of various best-practice guidelines for the integrationof FoX to large-scale computational chemistry simulation codes. By modifying NWChemto directly generate CML we avoid some of the potential difficulty with externalconverters discussed above. Any NWChem developer modifying the codebase will see,directly in the code being modified, the subroutine calls that generate the CMLdocument. This, in itself, alerts the developer of the potential for their changesto break the generation of CML (or, at least, for the potential to break someunknown feature of the code). This cue is absent in the case of external convertersthat parse an output file only designed for human consumption. If this hint todevelopers is insufficient the use of FoX provides a second line of defence againstbreakage of the CML output: such damage will result in the NWChem code failing tocompile or run simple test cases. The developer will thus be motivated to correctthe output immediately, hopefully before checking the change into their versioncontrol system. Again, this opportunity is absent in the case of the use of externalfile converters.

For comparatively simple applications the FoX_wcml interface should be sufficientlyclear to allow calls to be made directly from the main body of the simulation code.However, as the simulation code becomes larger it becomes important to isolate thecalls to the FoX library within a ‘glue’ layer (often a single Fortran90 module) within the application. This technique was adopted in NWChem. As istypical, the layer brings together groups of calls to FoX routines within ahigher-level interface adapted for the data structures used by the application andisolates the necessary (and often simple but tedious) data conversion tasks from theimportant numerical computation. This isolation helps minimise the possibility thatan unrelated change to the main numerical parts of the code would cause the CMLoutput to fail (for example, by attempting to generate an ill-formed documentresulting in FoX terminating execution). This can be particularly important if thecode is developed by a large team as it is possible that only a few developersunderstand the constraints of the FoX API and XML more generally. Placing the FoXcalls in an isolated module also makes it comparatively easy to make the generationof CML output a runtime or compile time option allowing, for example, some of themore egregious compiler bugs to be avoided when deploying on systems with a limitedchoice of Fortran compiler.

Once a computational chemistry code like NWChem produces CML data, tools are requiredto read in and analyse this data. One such tool is Avogadro, which was extended toread in the CML data files produced by NWChem and to display the molecular orbitalsusing the same library functionality written for other output formats. The majoradvantage of this approach over those previously taken is in the clear definition ofwhat each term means, and how it should be used. The file reader can also be muchsimpler as it makes use of standard C++ libraries to read in and scan the XMLdocument for the expected tags, allowing for a reader that is likely to continueworking as expected even as new output types are added to the XML document.

There are of course other options for data storage, with formats such as JSON beingused in an increasing number of projects for data exchange. The form and syntax ofJSON is much simpler than XML, allowing for smaller and simpler parsers but thereare also several drawbacks, which make XML a better choice for data exchange betweenloosely coupled components. JSON schemas are in their infancy, with no support fornamespaces and nascent validation tools, all of which can be very helpful whencomposing complex documents incorporating elements from several sources. This leadsto software that must generally be more tightly coupled to the data representationchosen in a particular implementation. This allows for XML based documents, such asCML, to compose multiple concepts such as scientific units defined by the widercommunity in a dedicated namespace composed with chemistry specific terms in the CMLnamespace, such as atomic positions and the ground state energy of the system. Dueto the similarities in base representation, round-tripping between CML and JSONrepresentations is not very difficult, and simple mapping schemes can be developedfor applications where JSON is preferred as the underlying storage mechanism orlightweight data exchange container in more tightly coupled systems. XML documentsretain a much richer semantic structure, which is also more readily converted toResource Description Framework (RDF) for further and more generalized consumption insemantic frameworks.

Not all important data produced by a computational chemistry simulation is suitableto be stored and fully expressed in an XML format. Examples include the generallylarge data files storing molecular orbitals, densities on a grid, or time evolutionfiles generated by molecular dynamics simulations. Work is underway to develop atwo-layer data infrastructure through the integration of the eXtensible Data Modeland Format (XDMF) [45] with the CML. Using XDMF allows semantically rich data, such as thecommon observables discussed above, to be stored in a searchable framework, whileall the large data (such as orbitals, trajectories, etc.) will be stored in thecompact HDF5 format [7].

### Chemical Markup Language for computational chemistry

Coupling diverse sets of data requires the development of a common language todescribe observables that should span experimental and computationaltechnologies. CML [18] provides a high level language for chemistry while the dictionariesand, to a lesser extent, the conventions, offer a starting point for thedevelopment of rich semantically enabled tools. Various fields, such ascomputational chemistry and NMR, XPS, and other experimental capabilities, aredeveloping conventions and dictionaries using CML. We believe that thesedictionaries can begin to be linked to form a more unified ontology [46, 47] to start to formally define relationships between terms in thedifferent fields of chemistry. Such an effort will provide the commonsemantically rich language needed to integrate diverse sets of data. However,the use of CML dictionaries is currently underdeveloped. Most of the currenttechnology only requires that dictionary references are used and that theyuniquely identify a particular concept. Increasingly these concepts are definedby entries in actual dictionaries and this is an important aspect of providingshared meaning but defining computationally accessible links between concepts isalso crucial. This requires both the links to be present in the dictionaries andtools to exploit these links, probably via a transformation to RDF, to bedeveloped.

Looking forward, one of the major challenges for the community will be to definea unified convention and dictionary for the electronic wave function. Thecurrent CML computational chemistry convention and dictionary for Gaussian basissets and effective core potentials is based on the XML format defined by theEMSL Basis Set Exchange. [48] Some work on defining a standard format for plane wave basis sets hasbeen done by Gygi et al. [15]. The EMSL Basis Set Exchange has the potential, especially whenexpanded to describe plane wave basis sets, to serve as a reference database onits own.

## Conclusions

We have developed an end-to-end use of semantically rich data in computationalchemistry within the Chemical Markup Language (CML) framework. NWChem was relativelyeasily modified to use the FoX library to produce well-formed and valid CMLdocuments that conform to recent conventions and are semantically rich. Draftdictionary entries as well as a proposed CML CompChem format for describingmolecular orbitals were developed and included in the FoX library. The FoX librarywas expanded to provide semantics that enable the scientific application torepresent the raw ASCII input file(s). A new CML reader developed for Avogadro wasused to read and visualize the NWChem computational chemistry CML data. We brieflydiscussed the need for a community-based development of a unified computationalchemistry convention and dictionary, and outlined some of the challenges to itsdevelopment.

The long-term goal is the development of a common language and data infrastructurefor the chemistry community that will enable machines to easily process datagenerated by computational chemistry tools. By clearly embedding the semantics ofthe document with the raw data we hope to limit or eliminate ambiguity when datadocuments are indexed, searched or otherwise processed and enable scientificdiscovery through simple and robust interfaces. We envisage a situation where aresearcher, prior to embarking on a calculation, enters key parameters in a searchtool, perhaps resembling the Google interface, and that this would return renderedand understandable representations of the output of previous calculations along withlinks to the published literature. By embedding the semantics inside the datadocument from their creation we remove the need for excessive inference of thesemantics by the indexing system. A useful side effect of this effort, demonstratedin this contribution, is the ease that the technology can be used to link existingsimulation and visualisation tools in the computational chemistry domain. At presentefforts are underway to integrate both experimental and NWChem’s computationaldata at the EMSL, a national user facility of the U.S. Department of Energy’sOffice of Biological and Environmental Research. Eventually, all CML data from thefacility’s computing capabilities will be available in a searchable dataarchive and researchers will be able to gain new scientific insight through accessand visualization of complex sets of simulation and experimental data.

## Appendix 1: Molecular orbitals dictionary entries

As discussed in the main text, in the process of adding CML output to NWChem wedeveloped a draft of dictionary entries with descriptions and a proposed CMLCompChem format for describing molecular orbitals. These dictionary entries anddescriptions will be integrated in the CML CompChem dictionary to a reference to thesemantic meaning of the concepts in CML documents that include the specification ofmolecular orbitals. This dictionary is already used in a range of applications thatrequire this information internally (e.g., for unit validation) or to provide humanreadable descriptions of the terms (e.g. to provide help text in documentstransformed into HTML). By being included in the dictionary, the new terms will alsobecome available on the web.

### Dictionary entries

Molecular Orbitals (dictRef: molecularOrbitals)

Definition: CML list container for all information related to one setof molecular orbitals.

Description: A set of wavefunctions describing all electrons in asystem of atoms.

Data Type: molecularOrbitals is of data type cml:list

Unit Type: molecularOrbitals has unit type unitType:none

Atomic Basis Descriptions (dictRef: atomicBasisDescriptions)

Definition: A cml array containing the descriptions of atomic basisfunctions.

Description: Atomic basis functions constructed from linearcombinations of Gaussian functions that describe atomic orbitals and form thebasis for the molecular orbital in the format < atom number:atom name, shell type>, and of the type xsd:string. Shell type refers to theangular momentum of the basis function (s, px, py, pz, dxx, dxy, dxz, dyy, dyz,dzz, etc. for cartesian basis functions and s, px, py, pz, d −2, d−1, d 0, d 1, d 2, etc. for spherical basis functions).

Data Type: atomicBasisDescriptions is of data type cml:array

Unit Type: atomicBasisDescriptions has unit type unitType:none

Molecular Orbital (dictRef: molecularOrbital)

Definition: A cml list container for one molecular orbital.

Description: Mathematical representation of the wavefunction of anelectron in a system of atoms described by a linear combination of atomic basisfunctions or atomic orbitals.

Data Type: molecularOrbital is of data type cml:list

Unit Type: molecularOrbital has unit type unitType:none

Orbital Energy (dictRef: orbitalEnergy)

Definition: Total energy of the molecular orbital.

Description: Energy of the electron described by the molecular orbitalin Hartrees.

Data Type: orbitalEnergy is of data type xsd:double

Unit Type: orbitalEnergy has unit type unitType:energy

Orbital Symmetry (dictRef: orbitalSymmetry)

Definition: Point group symmetry of the molecular orbital.

Description: Symmetry character of the molecular orbital within thepoint group symmetry of the molecule.

Data Type: orbitalSymmetry is of data type xsd:string

Unit Type: orbitalSymmetry has unit type unitType:none

Orbital Spin (dictRef: orbitalSpin)

Definition: Spin of the orbital.

Description: Spin symmetry of the molecular orbital as either“alpha” or “beta”.

Data Type: orbitalSpin is of data type xsd:string

Unit Type: orbitalSpin has unit type unitType:none

Orbital Occupancy (dictRef: orbitalOccupancy)

Definition: Occupancy of molecular orbital.

Description: Number of electrons occupying the molecular orbital. Thevalue will range from 0.0 to 2.0 electrons. When the orbital spin is defined aseither alpha or beta, the maximum occupation can be 1.0.

Data Type: orbitalOccupancy is of data type xsd:double

Unit Type: orbitalOccupancy has unit type unitType:none

Atomic Basis Function Composition of Molecular Orbital (dictRef: aoVector)

Definition: The cml array containing atomic basis functioncoefficients.

Description: A cml array of xsd:double containing the coefficients ofthe linear combination of atomic basis functions that describe the molecularorbital.

Data Type: aoVector is of data type cml:array

Unit Type: aoVector has unit type unitType:none

### Example of molecular orbitals

Below an example of the molecular orbitals CML format. Shown is the header andfirst two orbitals of the H2 molecule: 

## Appendix 2: Representing input data

In the process of adding CML output to NWChem we found that it is important toinclude a direct representation of the NWChem input parameters within the CML outputfor provenance. Although the CML Schema has sufficient flexibility to allow thisnone of the existing conventions contain a suitably defined mechanism for this task.In this appendix we outline a suitable microformat for the representation of Fortraninput files in computational chemistry. The intention is that this representationforms part of the CML CompChem convention but is sufficiently flexible to be reusedin other contexts. We note that a textural representation of input files does notoffer the same degree of semantic interoperability as including input data in afully marked-up format (i.e. as CML parameters with dictionary references andexplicit units). However, the task of recreating input files from such data is anon-trivial problem that has, thus far, received little attention. The microformatdefined here can be viewed as an essential intermediate step to allow a completerepresentation of the input data to be stored in the meantime. Applications makinguse of the microformat should additionally report input data semantically, as CMLparameters.

The microformat is designed to allow easy storage and retrieval of the content of oneor more files, or of data read from another file-like source (e.g. from standardinput). The aim is to make it possible to reconstruct the input using a simplestreaming parser without the need to build an in-memory representation of the CMLdocument (in order to handle cases of very large files) even if the XML document hasbeen modified (e.g. if the document has been subject to Canonicalization [49]). The approach is also designed to make data recovery using an XSLtransform straightforward. File metadata such as the original filename is alsoaccessible. We assume that only ASCII data must be stored, arbitrary binary filesare out of scope as are XML documents and non-ASCII textural data.

### Format for the representation of input data

This specification uses the namespaces and prefixes to indicate those namespacesas outlined in Table 1. Element names, and wherenecessary, attribute values, are given as QNames (i.e. in the formprefix:localName) and the prefix must be in scope and bound to the appropriatenamespace URI. The data format is defined as follows:

- The cml:module element may be used as a container for one or more input files. If used this outer module must have a dictRef attribute with the value ‘compchem:inputFileList’. There may be a title attribute giving this information in brief human readable form. Rationale: It is important to be able to easily locate input file data in large documents. By enclosing all such data in a single parent element with a uniquely defined dictionary reference a low-memory streaming parser can extract the data without the need for complex document manipulation.
- Data for each input file must be contained in its own cml:module element. Each such element must have a dictRef attribute with the value “compchem:inputFile” and may have a title attribute. It should be possible to convert the contents of each such module to a single ASCII file used as input to an atomistic simulation application without reference the rest of the XML document. Rationale: Some applications read input data from multiple input files. It must be possible to reconstruct each of these files.
- Each module element described in (b) should contain a single cml:metadataList element with zero or more number of cml:metadata child elements. These elements are intended to contain metadata that can be used help the (re)creation of the calculation’s input data. It is intended that a wider range of information could be conveyed in this way but we pay particular attention here to the file name of the input data file represented by the parent module. The file name should be stored as the content attribute of a metadata element with the name attribute being compchem:inputFileName. Rationale: Associated with each file are various items of metadata. While not all file-like objects will have a name where it is present the file name can be used to recreate the input data needed by the application (which, sometimes, must be contained in files with a fixed name).
- Each module element described in (b) should contain one or more cml:scalar child elements. These should have the attribute ‘dataType’ with the value ‘xsd:string’. Text content of these elements should each correspond to a single (XML encoded) line of the input file. All white space should be preserved but line-ending characters should be removed. The order of the lines in the input file must correspond to the document order of the cml:scalar elements as defined in section 5 of the XPath specification [50]. Empty lines should be represented by empty cml:scalar elements. Whitespace, including tabulation characters, multiple spaces and spaces at the start and end of lines should be preserved unaltered unless it is known that the application generating the CML document is insensitive to such changes. Rationale: This approach allows the location of each line of input via an XPath expression without the risk of modification due to normalisation of the document. Whitespace characters must be retained unaltered as, for some Fortran applications, the exact amount, form and location of such characters can be significant.

**Table 1:** **Namespaces and namespace prefixes used in the representation of inputfile data**

Prefix	Namespace URI	Description
cml	http://www.xml-cml.org/schema	Chemical Markup Language elements
convention	http://www.xml-cml.org/convention/	Standard Chemical Markup Language convention namespace
compchem	http://www.xml-cml.org/dictionary/compchem/	CompChem Dictionary namespace

The CML file shown in Figure 1 and included in theAdditional file 1 illustrates the use of thismicroformat.

### Reading and writing file data

A small number of new subroutines have been added to the FoX_wcml module topermit Fortran applications to easily create representations of their inputfiles in an output CML document using the format outlined above. Two methods areprovided with the most appropriate being dependent on the design of theapplication. In the first approach a single subroutine, cmlDumpInputDeck, iscalled with an array of file names as input arguments. In turn each file isopened, its contents are written to the CML document in the appropriate form,before the file is closed. The convoluted nature of file handling in Fortrancombined with the way that some applications read their input data means thatthis approach is not always available (for example, if the input file is heldopen for the duration of the calculation, or if data is read from standardinput) so an alternative interface with five subroutines (cmlStartInputDeckList,cmlStartInputDeckFile, cmlAddInputDeckLine, cmlEndInputDeckFile andcmlEndInputDeckList) is provided. The FoX documentation provides full details ofhow to use these two interfaces [51].

Finally, we provide a trivial example script to show one way of recreating inputfiles given a CML document. This uses XPath [50] and python and is provided in Additional file 1.

## Electronic supplementary material

Additional file 1: The following additional data are available with the online versionof this paper. Additional data file prop_h2o.cml is a fullexample of a validated CML file produced by the modified NWChem versiondiscussed in this paper, running the input file entitled prop_h2o.nw.The full output file prop_h2o.output is also provided. Additional datafile input_files_example.xml is a simple example of the proposed inputdata format described in Appendix 2 and produced with new features ofthe FoX_wcml library. Additional data file extract_input_files.py is astraightforward python script that can be used to reproduce input datafiles stored in a CML document following the format described inAppendix 2. (ZIP 86 KB)

Below are the links to the authors’ original submitted files for images.

Authors’ original file for figure 1

Authors’ original file for figure 2
