# Supplementary figures and images for: From data to analysis: linking NWChem and Avogadro with the syntax and semantics of Chemical Markup Language
Source: J Cheminform. 2013 May 24;5:25. doi: 10.1186/1758-2946-5-25 (PMC3764975; doi:10.1186/1758-2946-5-25)

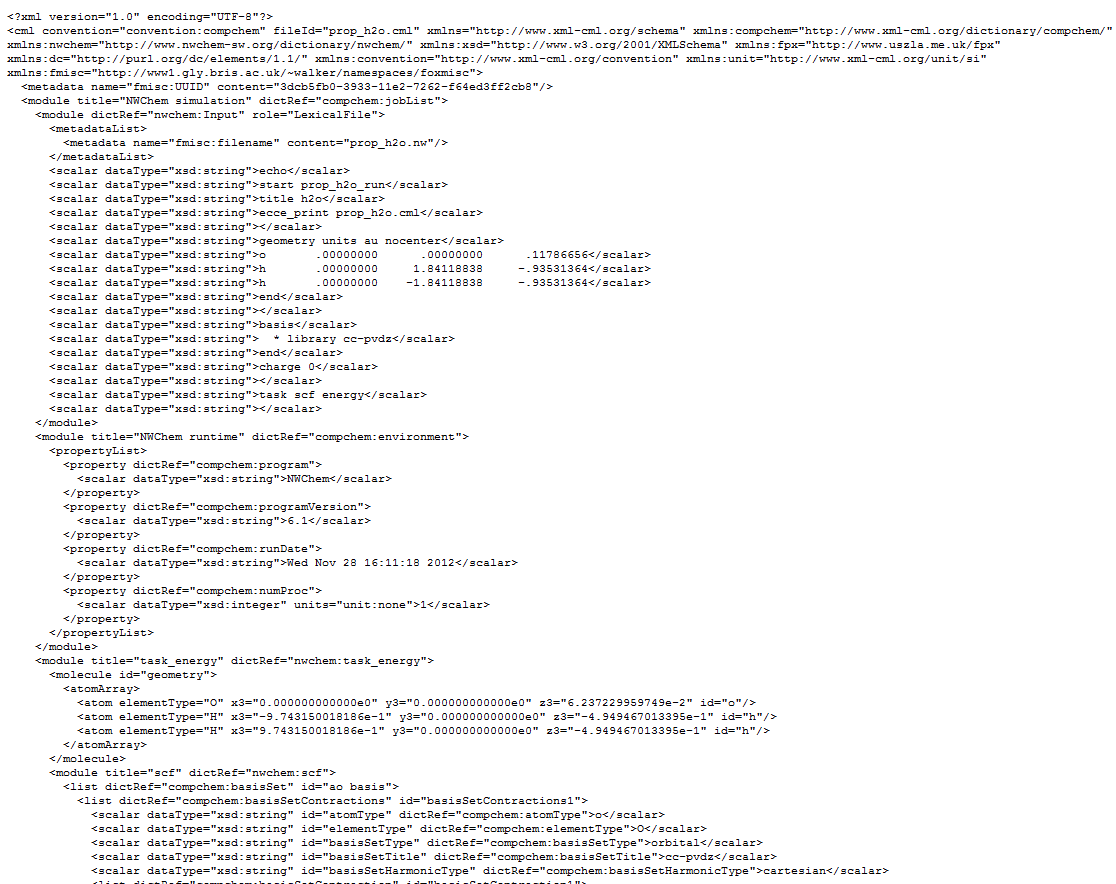

Supplement: Supplementary file 2 — Authors’ original file for figure 1 [file 13321_2013_470_MOESM2_ESM.png]

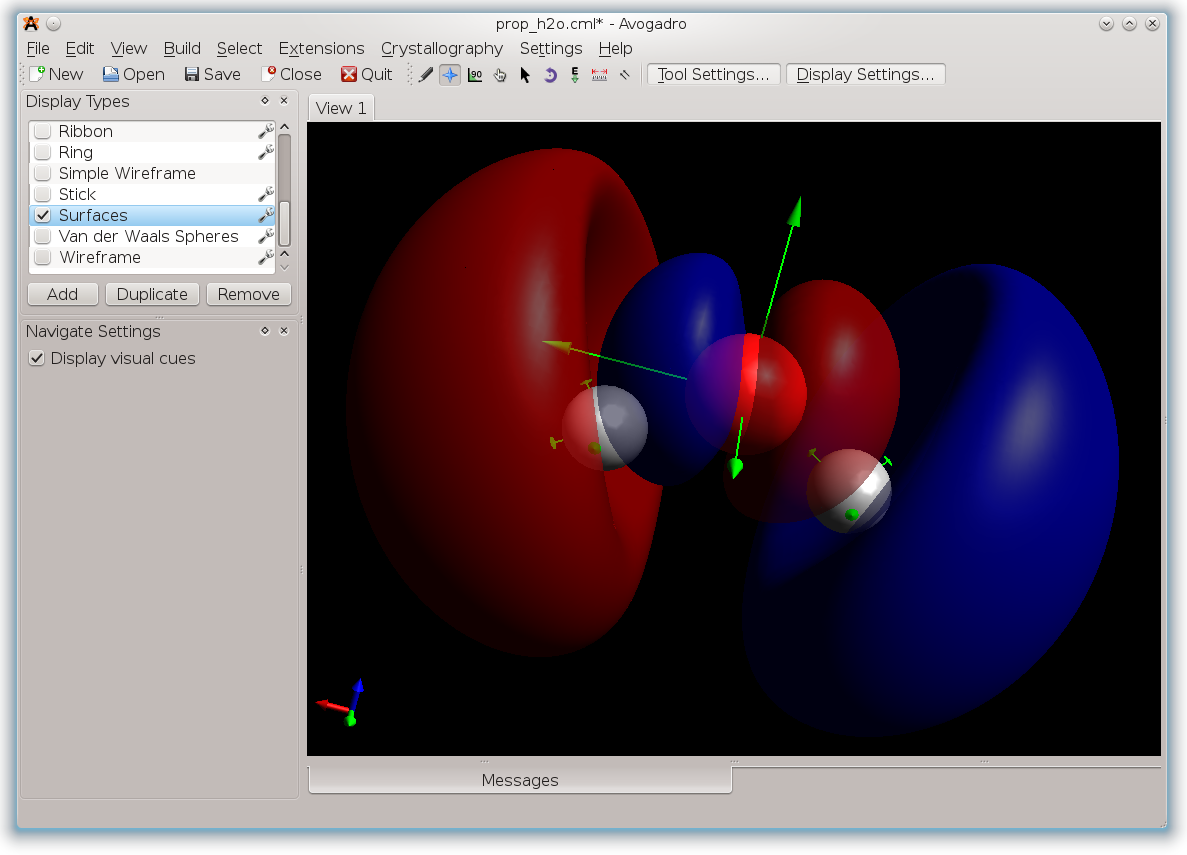

Supplement: Supplementary file 3 — Authors’ original file for figure 2 [file 13321_2013_470_MOESM3_ESM.png]
